# Medial patellofemoral ligament reconstruction and tibial tuberosity transfer can be used to successfully manage patellofemoral instability in the setting of trochlea dysplasia

**DOI:** 10.1186/s43019-023-00181-7

**Published:** 2023-04-27

**Authors:** Varun Dewan, Suribabu Gudipati, Joanna Rooney, Adam Lloyd, Sanjiv Chugh, Ejaz Mughal

**Affiliations:** 1 New Cross Hospital, Wolverhampton Road, Heath Town, Wolverhampton, WV10 0QP UK; 2 Stepping Hill Hospital, Poplar Grove, Hazel Grove, Stockport, SK2 7JE UK

**Keywords:** Patellofemoral instability, Trochleoplasty, Patella dislocation, Tibial tuberosity transfer, Medial patellofemoral ligament

## Abstract

**Background:**

Management of patella instability remains a challenge particularly in the presence of trochlea dysplasia. The aim of this study is to assess the recurrence rates of those with patellar instability who have undergone a combined tibial tuberosity transfer (TTT) and medial patellofemoral ligament reconstruction (MPFLR) in the setting of trochlea dysplasia.

**Methods:**

All skeletally mature patients who underwent combined TTT and MPFLR for recurrent patella instability were identified between January 2009 and December 2019. A retrospective review was conducted, with information regarding re-dislocation/subluxation and complications collected.

**Results:**

Seventy patients with a mean age 25.3 years were identified and evaluated. Thirteen patients were found to have low-grade dysplasia (Dejour A), with 57 patients having high-grade dysplasia (Dejour B/C/D). No patients in the low,grade dysplasia group suffered a recurrence of their symptoms, with four in the high-grade group suffering episodes of re-dislocation/subluxation. Three patients subsequently underwent a trochleoplasty, with the other patient managed successfully non-operatively. There were a total of 13 complications in 11 patients.

**Conclusions:**

A combined procedure of MPFLR and TTT can be used to manage patellofemoral instability even in the setting of trochlea dysplasia with a low rate of recurrence. Trochlea dysplasia, however, remains an anatomical risk factor for recurrence and patients should be counselled accordingly. The anatomical risk factors should be assessed in all patients to allow for the development of the most appropriate management plan, of which this combined procedure represents a potentially successful option.

**Level of Evidence:**

IV (Case Series).

## Introduction

A combined approach of a medial patellofemoral ligament reconstruction (MPFLR) combined with a tibial tuberosity transfer (TTT) has become increasingly popular for the management of patellofemoral instability (PFI) [1]. The medial patellofemoral ligament (MPFL) has been shown in both biomechanical and clinical studies to be the primary restraint against lateral patellar dislocation during early flexion prior to the patella’s engagement with the trochlear. [2, 3]. Indeed, there are those who advocate MPFLR or TTT in isolation for recurrent PFI [4–6]. However, neither of these procedures in isolation may be sufficient in counteracting the lateral force vector acting upon the patella at the time of dislocation. Furthermore, the role that these procedures play in the setting of trochlea dysplasia is also unclear. An increasing number of trochleoplasties are being performed for patellofemoral instability in the setting of trochlea dysplasia [7]. Whilst it has been reported that trochleoplasty surgery has a similar rate of complications when compared with other stabilization procedures [8], it is a highly complex procedure with a prolonged rehabilitation for the patient.

Therefore, the aim of the paper was to assess the recurrence rates of those with PFI who have undergone a combined procedure of TTT and MPFLR in the setting of trochlea dysplasia. We hypothesise that this combined procedure successfully stabilises the patella, even in the setting of high-grade dysplasia.

## Methods

### Study design and patient selection

All patients who underwent a MPFLR and TTT for PFI under the care of the senior author at our institution between January 2009 and December 2019 were identified. All skeletally mature patients with recurrent PFI, failed conservative management and a minimum follow-up of 6 months were included. Patients who were skeletally immature, had had any previous surgery to the knee or an additional procedure at the same sitting (e.g. trochleoplasty) were excluded. All patients underwent a standardized surgical technique and rehabilitation protocol.

### Operative technique

Each patient underwent surgical reconstruction using a standard protocol following completion of the World Health Organization (WHO) surgical checklist. Surgery was conducted either under a general anaesthesia and local anaesthetic infiltration or spinal anaesthetic. The patient was positioned supine on the table with thigh tourniquet and supports to hold the knee flexed to 90°. Tibial tuberosity osteotomy and transfer was carried out followed by MPFL reconstruction.

#### Tibial tuberosity transfer (TTT)

**Fig. 1:**
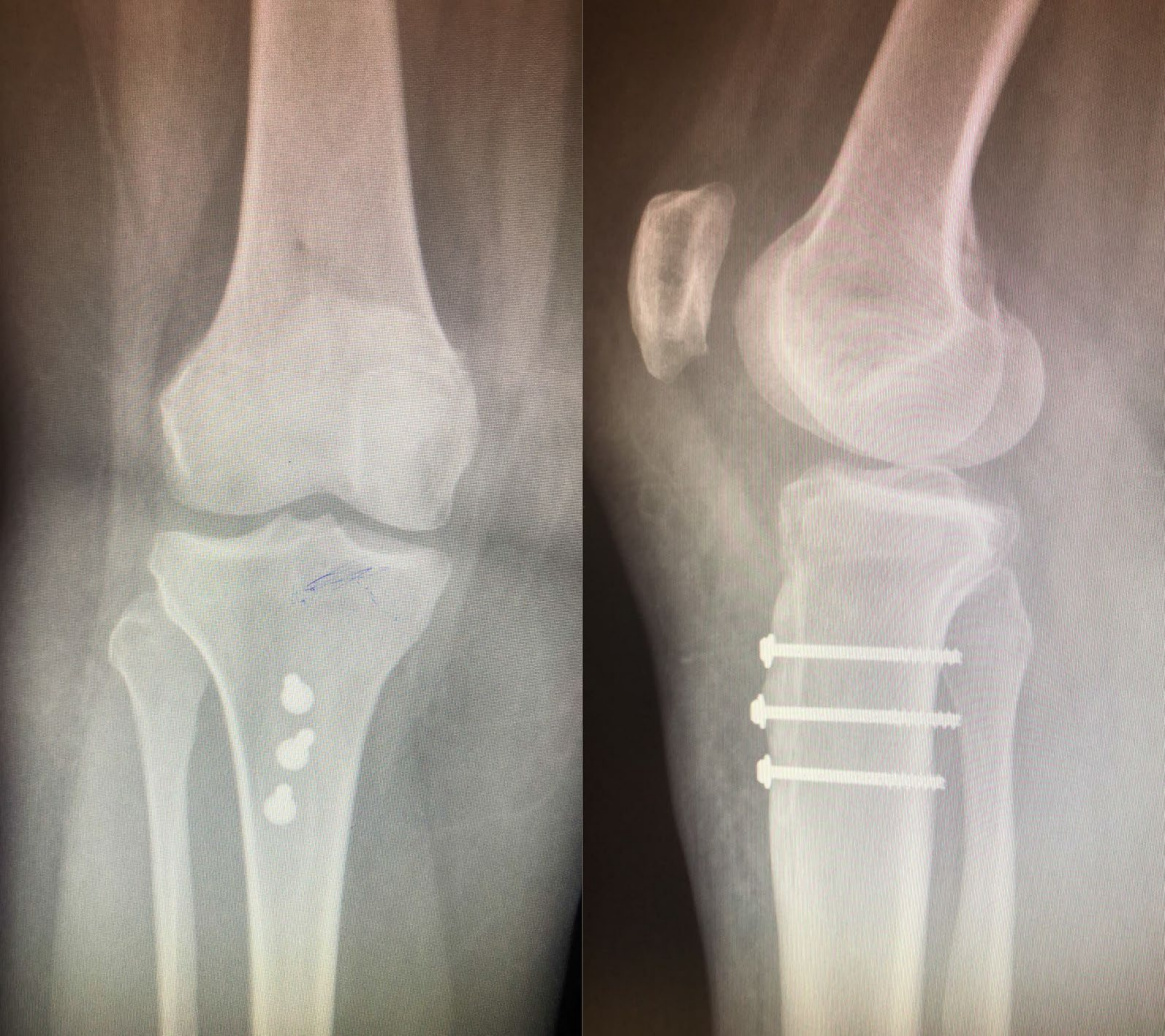
Antero-posterior and lateral post-operative plain radiographs demonstrating fixation of tibial tuberosity transfer

The TTT was performed using a previously published technique [9]. A single longitudinal incision is performed slightly medial to the tibial tuberosity. A further stab incision is made at the inferolateral border of the patella and extended proximally by 1 cm. Careful soft tissues dissection was carried out preserving the medial and inferior soft tissues. Thereafter, the tibial tuberosity osteotomy was undertaken using an oscillating saw from lateral to medial direction with the leg maintained in an internal rotation position. This is done just anterior to the anterolateral compartment muscles with the blade being angled upwards at the distal extent. Elevation of the osteotomy with an osteotome is performed carefully to ensure a distal soft tissue hinge remains intact. The knee is moved into extension and the osteotomy is medialised to establish a TTTG of 12 mm. Care is taken not to over medialise the position of the tibial tuberosity. The osteotomy is then stabilised with two or three 4.0 mm cannulated screws under image intensifier (II) guidance. The aim is to not breach the posterior cortex with the cannulated drill. The screws are inserted with a washer (Fig. 1).

#### Medial patellofemoral ligament reconstruction (MPFLR)

A 1.5 cm medial incision over the medial border of patella and two 5.5 mm helix anchors into the medial facet. An autologous graft (gracilis or semitendinosis) is inserted and secured on the medial side of the patella. A 2 cm incision is made over the MPFL attachment over the distal femoral condyle. An anterior cruciate ligament (ACL) pin is drilled into Schottle’s point [10] under II control. A soft tissue tunnel is created, and the graft is passed through it. The graft is then secured using a bioabsorbable biointerference screw in 30° of flexion in the femoral tunnel.

#### Post-operative rehabilitation

All patients were rehabilitated following a standardised in-house protocol in accordance with the physiotherapist advice. All patients are fitted with a hinged knee brace and were allowed protected weight bearing with the use of crutches for the initial 6 weeks with gradual development of quadriceps strength. Initial range of motion (RoM) was restricted to 90° flexion for 4 weeks. Once the tibial tubercle osteotomy has healed, RoM was progressively increased to full flexion.

Patients were followed-up for clinical and radiological assessment initially and then clinically alone after that. During the clinical visits, the senior author or a member of his team assessed each patient for any signs of instability. Plain radiographs were performed to evaluate the tibia tuberosity osteotomy healing. The development of any complications was also documented.

### Radiological assessment

All patients underwent magnetic resonance imaging (MRI) of the knee as part of their pre-operative assessment. These MRI scans were jointly reviewed by the senior author and a senior orthopaedic trainee to measure the tibial tuberosity–trochlea groove distance (TTTG), tibial tuberosity–posterior cruciate ligament distance (TT–PCL), patella height using patella trochlea index (PTI) and to classify the trochlea dysplasia.

### Outcomes

Details of post-operative complications, activity levels and recurrent episodes were also recorded.

### Statistical analysis

Statistical analysis was conducted using RStudio version 1.3.1093 (RStudio, PBC) with significance level of *p* < 0.05. Statistical analysis of the re-dislocation/subluxation and complication rates between the low grade (Dejour A) and high grade (Dejour B/C/D) dysplasia groups was performed. Wherever possible, descriptive statistics have also been used for analysis purpose.

## Results

**Fig. 2:**
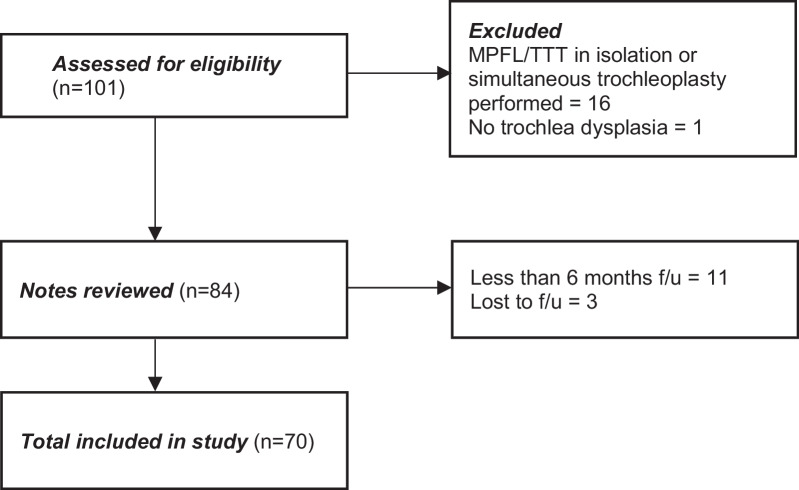
Study participant flow chart

Between January 2009 and December 2019, 101 patients (101 knees) underwent MPFLR + TTT for recurrent PFI under the care of a single surgeon at our institution. A total of 70 patients were included in the analysis (Fig. 2). The mean age of the patients was 25.3 years [standard deviation (SD) 9.5, range 14–51 years]. There were 24 males and 46 females. The mean follow-up was 2.0 years (SD 1.9, range 0.5–9.8 years). The mean TTTG was 18.1 (SD 3.8, range 14–27) and mean TTPCL was 27.9 (SD 5.2, range 21–44) (Table 1).

**Table 1:** Demographic data of included patients categorised by grade of trochlea dysplasia

	Low grade dysplasia	High grade dysplasia	*p*-Value
*N*	13	57	
Gender	8 F, 5 M	38 F, 19 M	0.73 n.s.
Side	3 R, 10 L	28 R, 29 L	0.09 n.s.
Mean age (years)	27.8	24.6	0.32 n.s.
Mean TTTG	17.2	18.4	0.31 n.s.
Mean TTPCL	27.2	28.0	0.57 n.s.
Mean PTI	0.29	0.32	0.44 n.s.

F: female; M: male; R: right; L: left; TTTG: tibial tuberosity–trochlear groove distance; TTPCL: tibial tuberosity–posterior cruciate ligament distance; PTI: Patella Trochlea Index; n.s.: not significant

Thirteen patients had evidence of low-grade dysplasia (Dejour A) and 57 had high-grade dysplasia (Dejour B/C/D). There was no statistically significant difference in TT–TG distance, TT–PCL distance or PTI between the two groups (*p* > 0.05).

Seventeen patients were known to participate in sports at varying levels prior to the onset of their symptoms. Following surgery, 58.8% (*n* = 10/17) were able to return to sporting activities following surgery.

There were a total of 13 complications in 11 patients (15.7%), with the most common being recurrence of patella instability (*n* = 4) and stiffness (*n* = 4).

None of the patients with low-grade dysplasia had any recurrence of their symptoms, whereas four patients (7.0%) in the high-grade dysplasia group experienced further subluxation and/or dislocation. Fisher’s exact test revealed that there was no difference (*p* = 0.43) in the incidence of the recurrence of patella instability between the low-grade and high-grade dysplasia groups following combined MPFLR and TTT. All patients who had a recurrence were female, with a mean age of 18.8 years. Three patients subsequently underwent a trochleoplasty and one patient was managed successfully non-operatively with physiotherapy. One patient had a non-union of their osteotomy which required further surgery and re-grafting.

There were no reported intra-operative complications. All complications are presented in Table 2.

**Table 2:** Complications following surgery

Complications	Low-grade dysplasia	High-grade dysplasia
Prominent hardware	0	2
VTE (DVT/PE)	1	1
Recurrence (dislocation/subluxation)	0	4
Non-union	0	1
Stiffness requiring MUA	0	4

VTE: venous thromboembolism; DVT: deep vein thrombosis; PE: pulmonary embolus; MUA: manipulation under anaesethetic

## Discussion

This study demonstrates that reconstruction of the MPFL and medialisation of the TT provides a successful modality of treatment for patients with recurrent PFI and increased TT–TG/TT–PCL, even in the setting of high-grade trochlea dysplasia, with a low rate of recurrence.

PFI is a highly complex condition owing to the numerous factors that contribute to its development. In those patients where surgical treatment has become necessary, the correctable factors should be addressed considering the anatomy of the PFJ and the biomechanical implications that they impart on its function. However, the current clinical evidence remains behind the biomechanical evidence. It is this biomechanical evidence that underpins the theoretical benefit of a combined MPFLR + TTT procedure. This has been corroborated by a recently published systematic review in which they found few high-quality clinical studies assessing the outcomes of this combined treatment [11]. Often these studies had small cohorts and there was a degree of heterogeneity amongst the study populations. The authors of this systematic review concluded that the current evidence basis was, therefore, inconsistent, as well as inconclusive, and could not be used to provide clear guidance. This study addresses some of these concerns and adds to the evidence of the effectiveness of this combined modality of treatment.

The MPFL is firmly established in the literature as the primary restraint against lateral patellar dislocation during early flexion which has been confirmed by numerous cadaveric biomechanical studies [12–15]. It provides up to 60% of restraint against lateral patella dislocation at varying degrees of flexion [2, 12, 16]. Therefore, unsurprisingly, MPFL reconstruction in isolation can achieve the necessary stability in patients with patellofemoral instability [4] with low recurrence rates [17]. Nevertheless, the evidence regarding performing isolated MPFLR in patients with high-grade dysplasia is not so conclusive. Hooper [18] reported a 100% recurrence rate in patients who underwent MPFL reconstruction in isolation with severe trochlea dysplasia (Dejour C or D) and worse outcome scores compared with those who had mild dysplasia (Dejour A or B). However, their definitions of mild and severe dysplasia are not in keeping with the literature and they did not specifically report the TT–TG for these patients. Conversely, Liu [19] demonstrated that MPFL reconstruction in the setting of trochlea dysplasia was associated with a statistically significant improvement in functional outcome scores with a recurrence rate of 2.5%. However, the mean TT–TG in their study population was 13.5 mm compared with 18.1 mm in this study. Chen [20] demonstrated the benefit of addressing the TT–TG in these patients. Their series of 25 patients with PFI, high-grade trochlea dysplasia and a mean TT–TG of 20.2 mm were treated successfully with MPFLR + TTT with no recurrence of symptoms at a mean follow-up of 36.8 months. Similarly, this study shows significantly lower recurrence rates when combining these two procedures for this cohort of patients. Therefore, in the presence of an elevated TT–TG and trochlea dysplasia, it is likely that an isolated MPFLR would have been insufficient in managing PFI.

Medialisation of the TT plays a role in reducing the lateral force vector acting upon the patella. Lateralisation of the TT has been shown to increase lateral patellar tracking and reduce patellar stability [21]. Methods of assessing the TT position exist but have limitations. The most commonly used method is TT–TG distance [22] but this suffers from inconsistently described parameters in the literature. There is no consensus on the value used to indicate the need for realignment surgery. A joint consensus statement from American Orthopaedic Society for Sports Medicine (AOSSM) and the Patellofemoral Foundation (PFF) [23], suggested that a TT–TG distance of > 20 mm was the upper limit of normal. However, it has also been reported that 20% of asymptomatic patients had a TT–TG > 20 mm [22]. Lower pathological values for TT–TG have also been reported in the literature. It has been shown that PFI is twice as likely to be seen in those with a TT–TG of > 13 mm [23]. The reported normal values have also been shown to less than the pathological values. Wittstein [25] described a normal TT–TG distance of 9.4 mm, whereas Pandit [26] described the normal distance to be 10 mm. Furthermore, there are concerns that TT–TG fails to take into consideration the tibiofemoral rotation. TT–PCL has been described as an alternative to TT–TG which is not influenced by any limb rotation and is a specific measure of TT lateralisation [27]. Its use has specifically been highlighted in patients with significant trochlea dysplasia [28]. All patients in our series had either an abnormal TT–TG value and/or abnormal TTPCL value. As a result, they underwent a TTT with the aim of reducing the lateral force vector acting upon the patella by reducing the TT–TG to 12 mm. Wagner [29] suggested that bony malalignment in patients undergoing isolated MPFLR was associated with a poor outcome. Indeed, some series have reported much higher recurrence rates of 28% in these patients [30]. Additionally, some concerns have also been reported in performing this procedure in younger patients in the presence of underlying trochlea dysplasia [31]. Trochlea dysplasia will likely remain a risk factor for recurrence in these patients. However, this study has shown that there is not a statistically significant increase in the recurrence rates of those with high-grade dysplasia compared with those who have low-grade dysplasia.

The limitations of our study are that it was a retrospective analysis of prospectively collected data. There is also no control group to provide comparative data and we do not have any formal outcome scores. Instead, we have used recurrence and return to sport as our markers of successful treatment. Long-term follow-up is also lacking, and it is important that future studies further look at the outcome of these patients to assess the true long-term risk of experiencing further instability. The key strength of this study is that all patients were managed under the care of a single surgeon with a standardised technique and rehabilitation programme.

## Conclusions

A combined procedure of MPFLR and TTT can be used to manage patellofemoral instability even in the setting of trochlea dysplasia with a low rate of recurrence. Trochlea dysplasia, however, remains an anatomical risk factor for recurrence and patients should be counselled accordingly. The anatomical risk factors should be assessed in all patients to allow for the development of the most appropriate management plan of which this combined procedure represents a potentially successful option.

## Data Availability

All data generated or analysed during this study are included in this published article.

## Abbreviations

PFI: Patellofemoral instability
MPFL: Medial patellofemoral ligament
MPFLR: Medial patellofemoral ligament reconstruction
TTT: Tibial tuberosity transfer
TT: Tibial tuberosity
MRI: Magnetic resonance imaging
II: Image intensifier
RoM: Range of motion
TT-TG: Tibial tuberosity–trochlea groove
TT-PCL: Tibial tuberosity–posterior cruciate ligament
PTI: Patella Trochlea Index
M: Male
F: Female
R: Right
L: Left

## Availability of data and materials

The datasets during and/or analysed during the current study available from the corresponding author on reasonable request and on approval from this host institution.
